# Genetic and functional diversity of double-stranded DNA viruses in a tropical monsoonal estuary, India

**DOI:** 10.1038/s41598-018-34332-8

**Published:** 2018-10-30

**Authors:** Vijayan Jasna, Ammini Parvathi, Abhinandita Dash

**Affiliations:** 1 CSIR-National Institute of Oceanography, Regional Centre, Kochi, 682 018 India; 2 Genotypic Technology, Bangalore, 560094 India

## Abstract

The present study illustrates the genetic diversity of four uncultured viral communities from the surface waters of Cochin Estuary (CE), India. Viral diversity inferred using Illumina HiSeq paired-end sequencing using a linker-amplified shotgun library (LASL) revealed different double-stranded DNA (dsDNA) viral communities. The water samples were collected from four stations PR1, PR2, PR3, and PR4, during the pre-monsoon (PRM) season. Analysis of virus families indicated that the *Myoviridae* was the most common viral community in the CE followed by *Siphoviridae* and *Podoviridae*. There were significant (p < 0.05) spatial variations in the relative abundance of dominant families in response to the salinity regimes. The relative abundance of *Myoviridae* and *Podoviridae* were high in the euryhaline region and *Siphoviridae* in the mesohaline region of the estuary. The predominant phage type in CE was phages that infected *Synechococcus*. The viral proteins were found to be involved in major functional activities such as ATP binding, DNA binding, and DNA replication. The study highlights the genetic diversity of dsDNA viral communities and their functional protein predictions from a highly productive estuarine system. Further, the metavirome data generated in this study will enhance the repertoire of publicly available dataset and advance our understanding of estuarine viral ecology.

## Introduction

Viruses are integral components of the marine microbial loop and are numerically most abundant biological entities in aquatic ecosystems^1–3^. They play significant roles in ecosystem functioning^4,5^. Apart from their direct impact on ocean biogeochemistry, viral infection significantly alters the structure and function of their prokaryotic and eukaryotic hosts^3^. Viruses exhibit high levels of host specificity in aquatic environments and are highly diverse in terms of their morphotypes and genotypes^6^. Despite their numerical abundance and ecological significance, very little is known about estuarine phage biodiversity and biogeography. Studies on marine viral diversity indicate that virioplankton diversity varies with seasonal and spatial variations in physico-chemical parameters^7,8^. In the past, viral diversity studies were limited compared to their microbial host community diversity studies as there is no single genetic element that is shared in all phage genomes like the bacterial 16S rDNA gene^9^. Further, the small size and low DNA content of viruses pose significant barriers to microscopic and molecular studies of diversity. However, metagenomics approach allows an in-depth characterization of molecular diversity, genome content, and structure of uncultured viruses, thereby delivering unique insights into the main viral families and their function in marine environments^10–13^.

Next generation DNA sequencing has been widely employed in the study of viral metagenomes (viromes) in different aquatic environments including fresh water^14,15^, oceans^10,16–19^ and reused wastewater^20^. It provides an in-depth and thorough analysis of genomics and proteomics of aquatic viruses from diverse habitats and decipher the role of viruses in aquatic ecology and biogeochemistry. This will give an estimation of the actual size of the viriosphere, information on virus infecting various hosts (both prokaryotic and eukaryotic hosts) and will subsequently contribute to the better understanding of the genetic diversity of life. However, two-thirds of the genes within the reported metavirome cannot be assigned a biological function or taxonomic affiliation, which makes viral species distribution similar among viromes from different environments^21^. In aquatic systems, the major factors influencing viral community structures are the trophic status, microbial diversity and their abundance. The viral communities change in response to various environmental factors such as temperature, dissolved oxygen, and chlorophyll *a*^22^. The viral communities from near shore waters, sediments and deeper oceans have been examined, but only a few reports have addressed the genomes of virioplankton in highly productive estuarine systems.

Earlier reports on viral ecology point out a unique distribution of viruses in the Cochin estuary (CE) in response to changes in host abundance and salinity^23^. The viral-mediated prokaryotic mortality showed significant seasonal variations with maximum viral-mediated mortality during the dry pre-monsoon season (PRM)^24^. The viral shunt was highest in the PRM, especially in the mesohaline regions of the estuary^24^. Previous studies on diversity of phytoplankton and zooplankton suggest that the species diversity, richness and evenness were high during the dry pre-monsoon season in CE due to high water temperature and reduced river run off^25,26^. Based on this, we chose to study the viral diversity during the dry pre-monsoon season from four different salinity regimes in the CE. We hypothesize that the spatial variations in viral activity could be due to variations in viral communities in the estuary. The present study was carried out to understand the genetic and functional diversity of viruses during the pre-monsoon season when the viral activity is high. This study presents a detailed report on the metaviriome analysis from a highly productive estuarine system.

## Results

### Environmental Parameters

The samples were collected during the dry pre-monsoon period when the system was highly stratified. The four sampling locations lie in a longitudinal transect between 76°15′ to 76°25′E and in a latitudinal transect between 9°30′ to 10°10′N, along the Cochin estuary in India (Supplementary Fig. 1). Temperature and salinity values ranged from to 31 °C to 32.78 °C and 4.6 to 30 ppt respectively (Table 1). The maximum temperature was noted at PR4 (32.78 °C) and minimum at PR3 (31 °C). The 4 stations exhibited different salinities with PR1 falling in the euryhaline region (29.9 ppt), followed by PR2 (23.36 ppt), PR3 (16.5 ppt), and PR4 (4.7 ppt). At all the locations, the highest/lowest salinity coincided with the highest/lowest tidal amplitude. The average tidal height in the inlet region (PR1) was 0.7 m, which decreased toward the upstream region (PR4) (0.5 m). The Chlorophyll *a* ranged from 2.36–12.69 mg/m^3^ with a maximum Chl *a* (33.11 mg/m^3^) at PR2 and least at PR4 (2.36 mg/m^3^). The dissolved oxygen concentration was high during the study period throughout the estuary with higher values at PR4 (5.51 µM). The spatial differences in NO_2_, NO_3_, PO_4,_ and SiO_4_ were significant between all the stations (Table 1).

**Table 1:** Comparison of physicochemical and biological parameters in stations PR1, PR2, PR3 and PR4.

	PR1	PR2	PR3	PR4
VA (10^7^ VLPs/ml)	3.21	2.54	2.72	1.93
PA (10^6^ Cells/ml)	3.03	2.78	2.96	3.06
TVC (10^6^ Cells/ml)	1.21	0.987	0.975	1.07
VPR	10.59	9.13	9.18	2.06
Chl *a* (mg m^-3^)	3.2	12.69	10.68	2.36
Phe (mg m^-3^)	5.23	3.20	2.35	8.12
Salinity (ppt)	29.99	23.36	16.45	4.69
Temperature (°C)	31.2	31.5	31	32.78
PH	7.92	7.74	7.45	7.36
DO (mg L^-1^)	3.57	3.88	3.55	5.51
NO_2_ (μM)	1.32	0.23	2.57	0.2
NO_3_ (μM)	13.14	12.1	3.72	1.4
NH_4_ (μM)	5.24	3.51	8.66	6.53
PO_4_ (μM)	2.03	2.3	10.89	0.223
SiO_4_ (μM)	14.44	30.1	21.13	75.33

Abbreviations used, Temp-Temperature, DO – Dissolved Oxygen, NO_2 –_ Nitrite, NO_3_ – Nitrate, PO_4_ – Phosphate, SiO_4_ – Silicate, Chl. *a* – Chlorophyll *a*, PA- Prokaryotic abundance, VA-Viral abundance, VPR- Virus to prokaryote ratio, Chl-Chlorophyll *a* and Phe-Pheophytin.

### Biological Parameters

**Figure 1:**
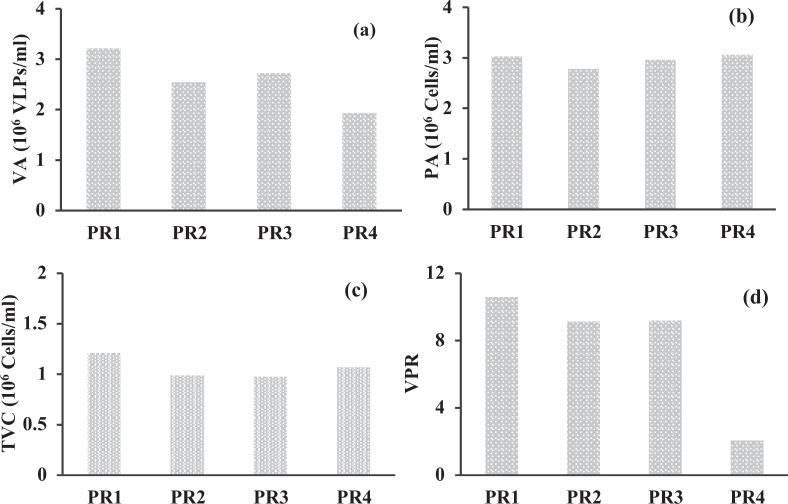
Represents (**a**) Viral abundance (VA), (**b**) Prokaryotic abundance (PA), (**c**) Total viable prokaryotes (TVC) and (**d**) Virus to prokaryote ratio (VPR). The stations are represented in the X axis and the red variables in Y axis.

The abundance of prokaryotes and viruses exhibited a distinct spatial pattern in their distribution (Table 1). The viral abundance (VA) ranged from 1.9–3.2 × 10^7^ virus-like particles per mL (VLPs mL^−1^) whereas the prokaryotic abundance was one order of magnitude lesser (2.7–3.1 × 10^6^ cells mL^−1^) than the viral abundance. The values of VA, PA and TVC in the study were comparable with previous reports from the Cochin estuary (CE)^23,24^. The virus to prokaryote abundance ratio (VPR) was used to examine the relationship between the viral and prokaryotic populations. The VPR ranged from 2.1 to 10.6. The highest and lowest VPR were recorded in the high saline and freshwater regions of the estuary, respectively (Fig. 1).

### Analysis of sequences in the Cochin estuary

The denovo assembly generated 1,18,872 contigs at PR1, 1,89,912 contigs at PR2, 2,86,178 contigs at PR3, and 3,02,030 contigs at PR4. The contigs with length >=300 bp were considered for further downstream analysis. The contig lengths for the annotations of each sample are illustrated in Table 2. The viral diversity of the four estuarine samples were different, but it was interesting to see that the PR1 inlet station had the highest alpha diversity comprising 172 unique viruses, followed by PR3 (163 unique viruses), PR4 (158 unique viruses), and PR2 (129 unique viruses), respectively. Detailed information regarding sequencing metadata, assembly metrics, and BLASTx searches is summarized in Table 2.

**Table 2:** (a) Overall statistical analysis of sequences from four different stations PR1, PR2, PR3 and PR4.

(a) Overall analysis statistics	PR1	PR2	PR3	PR4
Total raw reads	8812424	9001277	10997731	10789527
Total processed reads	6809780	7274859	8494976	8146808
Scaffolds(>=300 bp)	118872	189912	286178	8146808
unannotated scaffolds	116997	189606	285743	301636
Total number of unique viral populations	172	129	163	158
**(b)Assembly QC Result**				
Contigs Generated:	1,18,872	1,89,912	2,86,178	3,02,030
Maximum Contig Length:	9,27,566	6,68,508	11,84,905	4,51,237
Minimum Contig Length:	300	300	300	300
Average Contig Length:	835.6 ± 5,274.2	779.4 ± 3,758.7	862.5 ± 4,099.7	798.3 ± 3,020.2

Number of total reads, number of processed reads, unique viral population etc. are represented for the four stations. (b) Represents the Assembly QC results from four different stations PR1, PR2, PR3 and PR4.

### Taxonomic diversity of viruses in the Cochin estuary

**Figure 2:**
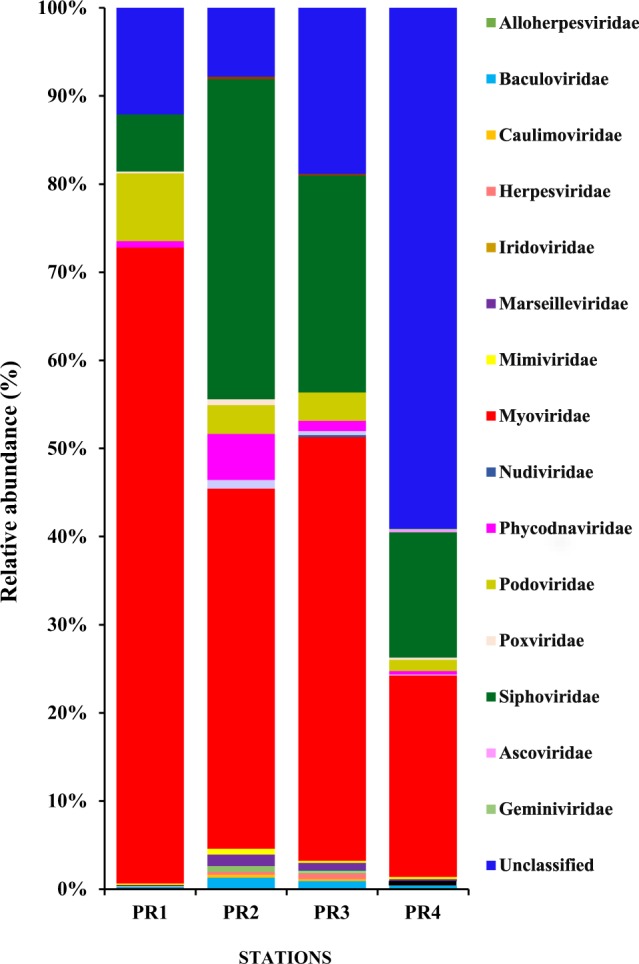
Taxonomic composition of viromes in the four stations in the Cochin estuary is represented as stacked bar charts. Stations PR1, PR2, PR3 and PR4 are plotted on the X axis and relative abundance (percentage, %) of different viral families are represented in the Y axis.

The taxonomic distribution of the assignable sequences greatly diverged among the four estuarine samples. Based on the relative occurrence values (GG PLOT2 R-package) at the family level, 18 families of double-stranded DNA (dsDNA) viruses were obtained (Fig. 2, Supplementary Figs 2 and 3). The order *Caudovirales*, known as tailed bacteriophages, was the most dominant order among the viruses annotated in this study in all the stations. *Myoviridae* was the most abundant family in all four stations, with highest dominance at PR1 (72.10% at PR1, 40.84% at PR2, and 48.05% at PR3) and was the least abundant in the freshwater region (23.69% at PR4). *Siphoviridae* was the second most abundant family, constituting about 6.45% at PR1, 36.27% at PR2, followed by 24.6% at PR3, and 20.43% at PR4. Family *Podoviridae* was least represented (7.68%, 3.26%, 3.21%, and 2.5% at PR1, PR2, PR3, and PR4, respectively) compared to *Siphoviridae* (Supplementary Fig. 3a–d). However, more than 60% of the sequences at PR4, 18.8% at PR3, 7.8% at PR2, and 12.1% at PR1 did not show any similarity to any of the known virus species, which were considered as viral dark matter.

**Figure 3:**
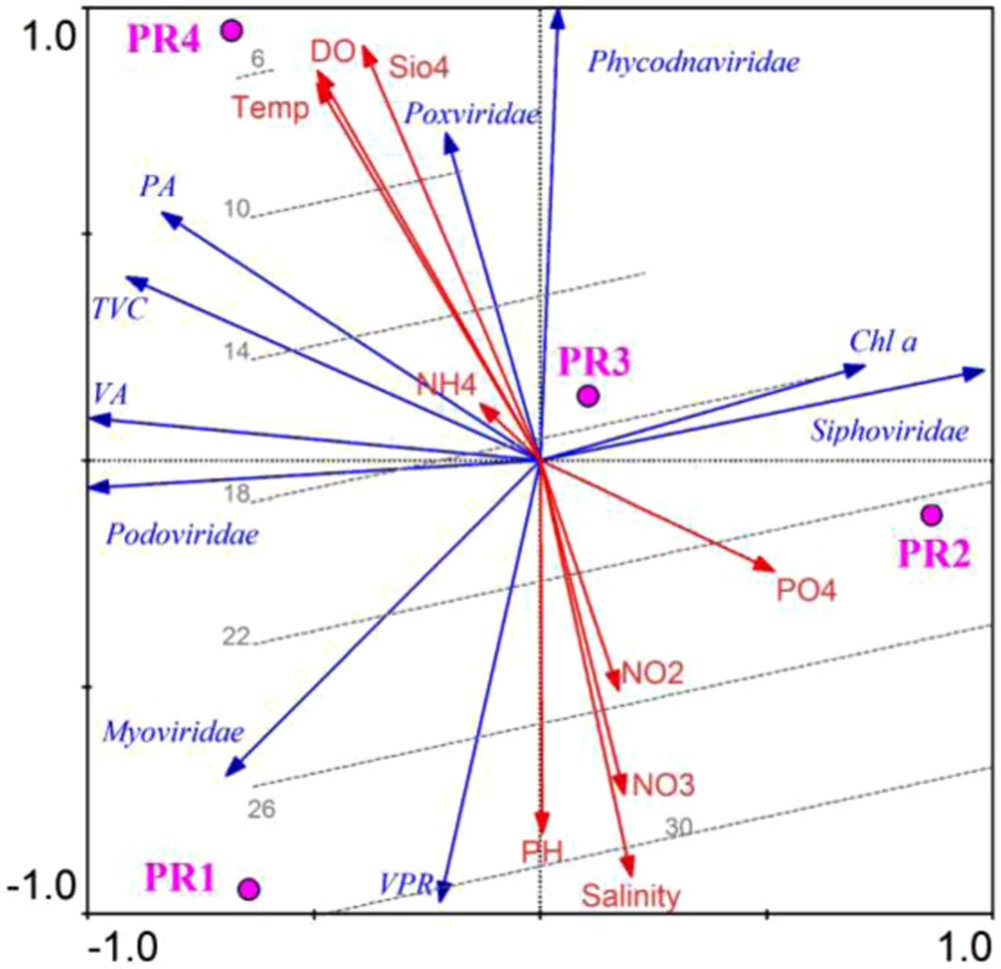
RDA triplot representing the distribution of viral families (*Myoviridae*, *Siphoviridae*, *Podoviridae*, *Poxviridae*, *Phycodnaviridae)* along with physicochemical (red lines) and biological parameters (blue lines) in the Cochin estuary (CE). Salinity contours (dotted lines) are overlaid on the triplot to show the interrelationships between physicochemical and biological parameters on distribution of viral families in the CE. The stations PR1, PR2, PR3 and PR4 are represented as pink filled dots.

In the present study, the dominant families were similar in all four locations. An RDA plot was generated to understand the factors influencing the abundances of viruses and prokaryotes, TVC, and distribution of major families such as *Myoviridae*, *Podoviridae*, *Siphoviridae*, *Poxviride*, and *Phydodnaviridae*, along with various physico-chemical variables **(**Fig. 3). The salinity was superimposed onto this RDA plot to determine the impact of salinity on the distribution of different viral families in the CE. Virus to prokaryote ratio (VPR) was high in the euryhaline region of the estuary. There were spatial variations in the relative abundances of dominant families, with *Myoviridae* being the most dominant family in all locations. However, the relative abundance of *Podoviridae* and *Siphoviridae* varied in different regions of the estuary. The highest abundance of *Siphoviridae* was recorded at PR2 where the salinity was 23 ppt, while the lowest abundance was recorded in the euryhaline region (PR1) of the estuary. In contrast, *Podoviridae* was more abundant in the euryhaline region (PR1) and least abundant in the freshwater region (PR4) of the estuary.

There was also smaller representation of sequences in the four estuarine viromes belonging to families *Baculoviridae* (insects and other arthropods), *Marseilleviridae* (amoebal viruses, also found in humans), *Mimiviridae* (gaint marine protists viruses), and *Phycodnaviridae* (large double stranded DNA viruses that infect marine or freshwater eukaryotic algae). However, minor families showed unique spatial distribution, for e.g. *Nudiviridae* (viruses of insects and marine crustaceans) in PR3, and *Ascoviridae* (viruses of invertebrates) in PR4. *Herpesvirales*, responsible for causing diseases in animals and humans, were also found in the CE. However, in lower resolution, *Poxviridae*, *Alloherpesviridae*, and *Iridoviridae*, were also present (Supplementary Fig. 3)

**Figure 4:**
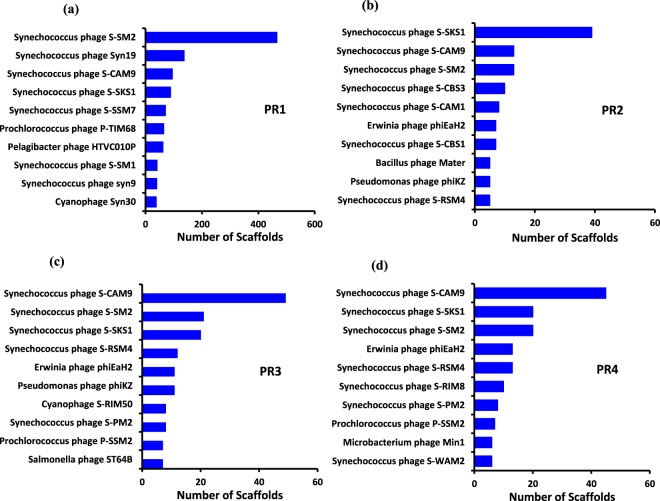
Barplot representing the abundance of the 10 most common virus species present in the metavirome at the stations, PR1, PR2, PR3 and PR4. The number of scaffolds is represented in the X axis and the species is represented in the Y axis.

**Figure 5:**
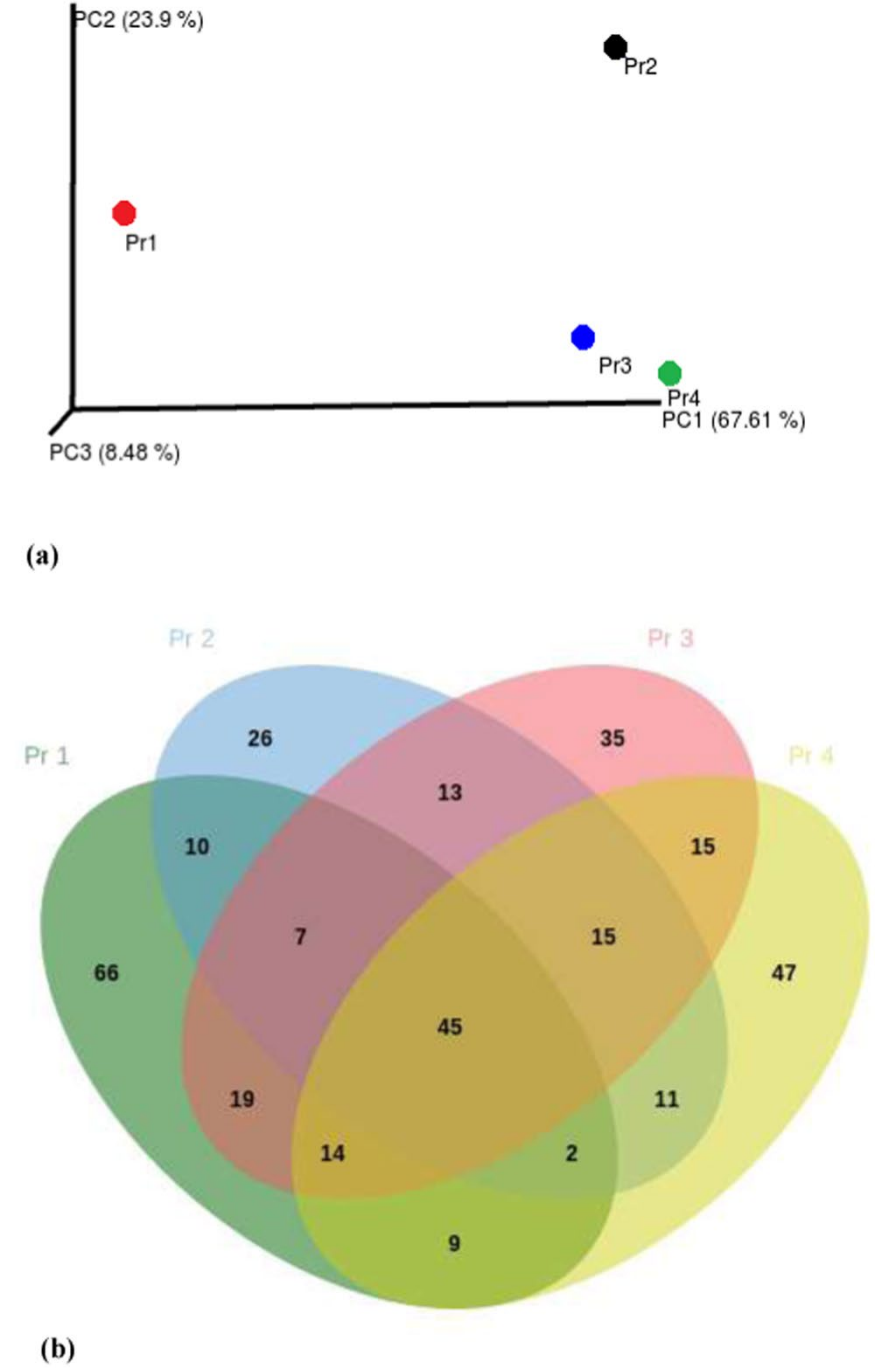
(**a**) Principal component analysis (PCA) triplot representing the distribution metavirome from 4 stations based on the abundance of sequences (**b**) Venn diagram representing the number of unique and shared viral sequences at four different stations PR1, PR2, PR3 and PR4 in the Cochin estuary.

The *Synechococcus* phage was the most dominant phage at all the four locations contributing to 70.3% in PR1, 42.8% in PR2, 41.1% in PR3, and 39.6% in PR4, respectively. The other major phages at PR1 were *Prochlorococcus* phage (8.5%), *Cyanophage* (6.9%), *Pelagibacter* phage (4.5%), and *Pseudomonas* phage (1.9%), whereas at PR2, *Pseudomonas* phage (5.9%), *Cyanophage* (3.9%), *Bacillus* phage (3.6%), and *Mycobacterium* phage (3.6%) were dominant. Similarly, in PR3, *Pseudomonas* phage (7.1%), *Cyanophage* (4.41%), *Prochlorococcus* phage (3.2%), and Mycobacterium phage (3%) were dominant, whereas, in PR4, *Pseudomonas* phage (6.3%), *Erwinia* phage (4.3%), *Mycobacterium* phage (4.3%), *Prochlorococcus* phage (3.8%), and *Cyanophage* (3.6%) were dominant (Fig. 4). The maximum hit reads of the top ten viruses are represented in Fig. 4. *Synechococcus* phages *S-SM2* and *S-SKS1* were dominant in PR1 and PR2, respectively, whereas *Synechococcus* phage *S-CAM9* was dominant in both PR3 and PR4 (Fig. 4 and Supplementary Fig. 4). There were 66 unique viruses in PR1, 26 in PR2, 35 in PR3 and 47 in PR4. A total of 45 viruses were shared by all the four stations **(**Fig. 5).

### Functional predictions of viral proteins

The functional prediction of the metavirome of four estuarine samples was performed based on structural and functional genes. The total percentage of annotated proteins was 25.32% for PR1 but was comparatively low for other stations (12.03%, 11.4%, and 15.41% for PR2, PR3, and PR4, respectively). The functional categories were assigned mainly to three major groups such as molecular, biological, and cellular functions.

**Figure 6:**
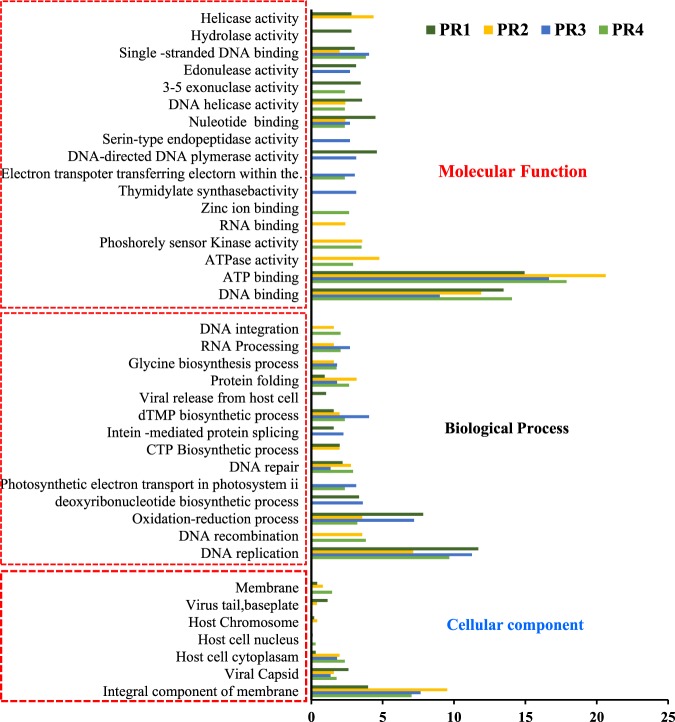
Functional diversity of the viral sequences based on the protein prediction. The panels a, b, c and d represent the predicted viral proteins in stations PR1, PR2, PR3 and PR4 respectively.

The major molecular functions of viral genes included DNA binding (13.47%, 11.9%, 14.08%, and 9.01% for PR1, PR2, PR3, and PR4, respectively of annotated functions), followed by ATP binding (14.94%, 20.63%, 17.89%, and 16.68% for PR1, PR2, PR3, and PR4, respectively). Additionally, genes involved in activities such as ATPase, DNA polymerase, hydrolase, helicase activity, nucleotide binding, endo and exo nuclease activity, RNA binding, nucleic acid binding, etc. were also detected in different viral groups. The major biological functions were DNA replication (contributing to 11.7%, 7.14%, 9.68%, and 11.26% for PR1, PR2, PR3, and PR4 respectively of annotated functions), followed by oxidation reduction process (7.84%, 3.57%, 3.23%, and 7.21% for PR1, PR2, PR3, and PR4, respectively of annotated functions). The other biological functions performed by viruses included DNA repair, DNA integration, RNA processing, protein folding, glycine biosynthetic process, dTMP biosynthetic process, and photosynthetic electron transport in photosystem II. The major cellular functions included genes involved in integral component of membrane, viral capsid, host cell cytoplasm, host chromosome, and host cell nucleus (Fig. 6).

### Spatial changes of viruses in CE

Heat map analysis of viral genes indicated that the spatial variation in the dominant families did not demonstrate significant changes, but the less abundant viruses varied with stations (Supplementary Fig. 5). The most dominant viruses were cyanophages such as *Synechococcus* phage in all the stations, followed by *Prochlorococcs* phage in PR1, *Pseudomonas* phage in PR2 and PR3, and *Erwinia* phage in PR4. *Puniceispirillum* phage, *Chrysochromulina ericina virus*, and *Yellowstone lakemimivirus* were present only in PR1. *Xanthomonas citri* phage, *Simbu virus*, *Azospirillum* phage were present in PR2, *Orgyia pseudotsugata*, *Acanthamoeba polyphaga mimivirus*, *Actinoplanes* phage were present in PR3, and *Bromus catharticus striate mosaic virus*, *Choristoneura occidentalis*, and *Cyprinid herpes virus* were present in PR4. The PCoA and heat map analysis showed that PR1 and PR2 formed a different cluster from PR3 and PR4 (Fig. 5, Supplementary Fig. 5).

### Global comparison of viral metagenomes from different environments

**Figure 7:**
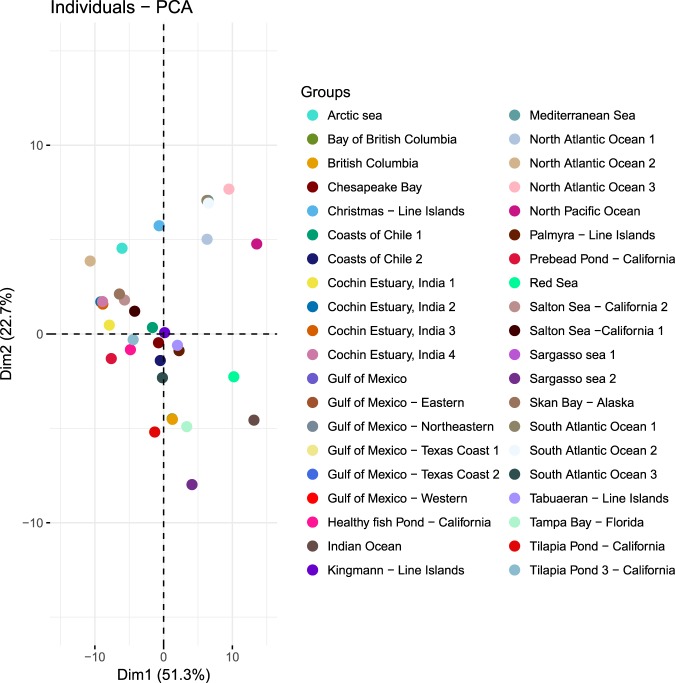
Principal Component Analysis (PCA plots) of global comparison of viromes from different types of environmental samples using MG-RAST. The metaviriome included datasets from Atlantic ocean (MG RAST id; 4722276.3 to 4722285.3), Gulf of Mexico (4440304.3, 4441623.3 to 4441629.3), Salton Sea (4440327.3, 4440328.3), Sargasso Sea (4441624.3, 4440322.3), Arctic sea (4440306.3), Line Islands 4440036.3, 4440038.3, 4440040.3, 4440280.3), Western Sea, Korea (4464802.3, 4464804.3, 4464805.3) Indian Ocean (4722282.3), Red Sea (4722283.3) bays (4440102, 4440330.3, 4440102.3), and Fish pond (4440424.3, 4440412.3, 4440439.3, 4440414.3).

The metavirome from the CE was compared with metaviromes from both similar and dissimilar environments, including marine, estuarine, fresh water, hyper saline water, fish pond water, and waste water based on previously published datasets. Marine and estuarine samples included metavirome data from the Chesapeake Bay, Tampa Bay, Skan Bay, Bay of British Columbia, Red Sea, Sargasso Sea, Mediterranean Sea, Arctic Sea, Gulf of Mexico, Indian Ocean, Atlantic Ocean, and Pacific Ocean. Overall, we found that global metaviromes showed a major proportion of Myoviridae, followed by Siphoviridae and Podoviridae. Within the dsDNA viruses, members of rare taxonomic groupings such as the genera Alloherpesviridae, Alphabaculovirus, Betabaculovirus, Betanudivirus, Cavemovirus, Chordopoxvirinae, Herpesviridae, Chlorovirus, Chordopoxvirinae, Phaeovirus, Prasinovirus, Prymnesiovirus, and Ranavirus were detected in the CE. These minor groups were not detected in other MG-RAST metavirome sequence datasets used for comparison. The principle component analysis (PCA) revealed the viral communities consistently clustered according to their similarity, significantly separating with dissimilar viral communities **(**Fig. 7). The metavirome from the Cochin estuary were quite similar to each other. The PCA indicated that CE viromes were closely related to metavirome of Salton Sea, California and Skan Bay. Salton Sea is a eutrophic lake with salinities ranging from freshwater, brackish to hypersaline waters. This lake is also characterized by high nutrient loading resulting in algal blooms throughout the year. Metavirome from coastal California and Skan Bays also represented from nutrient rich coastal environments. The results suggest similarity in metavirome data from similar environments.

## Discussion

The biogeography of specific viruses or viral sequences is widely unknown. As a result of methodological limitations, only a limited number of studies, especially from estuarine environments are reported for viral metagenome. The present study investigates the viral diversity using the metavirome approach with a linker amplified shotgun library (LASL). In this method, the sheared viral DNA is ligated with an adapter or linker, which can ligate only to double –stranded DNA (dsDNA). Hence LASL is used for the amplification of double stranded DNA and has been widely employed for deciphering the marine viral metagenomes^11,12,27^. However, another method, known as the multiple displacement amplification (MDA) has also been applied in marine viral metagenome studies to specifically amplify single stranded viruses. Therefore, these two amplification methods have been employed in metaviriome studies to reveal different aspects of viral diversity. Majority of metavirome studies have focused on ds-DNA containing viruses, although other nucleic acid type viruses are also studied^28–30^. However, since MDA has several disadvantages such as the production of biases through the formation of chimeras^31^ and quantitative biases^32^, we resorted to the use of LASL for our study, which gives information on dsDNA viruses alone. Further studies should be performed to estimate the relative abundance of ssDNA and dsDNA by using unamplified viral metagenomes or by using unbiased amplification methods in this estuary.

The results of our study illustrate that over 80% of the metavirome sequences were similar to the tailed bacteriophages belonging to the order *Caudovirales*. Previous reports on aquatic viriomes suggested high prevalence of *Caudovirales* in the Sargasso Sea^33^, Iquique- Chile^34^, Lake Pavin and Lake Bourget in France^35^. However, the Tara Oceans Expedition^36^ and Southern Indian Ocean Expedition^37^ report the dominance of non-tailed viruses using metagenomic approaches. *Myoviridae* was the most dominant family throughout the CE, followed by *Siphoviridae* and *Podoviridae*. There were spatial variations in the relative abundance of these major families in the CE. The dominance of *Myoviridae* indicated that bacteria were the most important host species. This correlates with our earlier reports from the CE on the high prokaryotic abundance, high contribution of viral lysis/viral shunt to the dissolve organic carbon pool during the high saline pre-monsoon season^24^. High abundance of bacteriophages in CE suggests that they are not only predators of bacteria but also play significant roles in the ecology and biogeochemistry of this ecosystem^24^. The rich organic pollutants in this estuary support high bacterial respiration and a low bacterial growth efficiency. An increase in ionic strength during the pre-monsoon season also alters the organic matter-inorganic matter association/interaction. The resultant increase in the bioavailability of the organic matter increases the bacterial metabolism and bacterial respiration^5^. Thus, viral lytic activity, being host-dependent, is high during the highly saline pre-monsoon season in the CE. This substantiates the dominance of *Myoviridae*, the virulent broad host-range viruses, throughout this estuary. Our results were comparable with the viral diversity at the Chesapeake Bay, where *Myoviridae* was the most dominant family followed by *Podoviridae*, and *Siphoviridae*^12^.

The spatial variation among dominant families seemed to be strongly influenced by salinity regimes in the CE. *Myoviridae* was dominant at all the locations, *Podoviridae* was relatively more abundant in the euryhaline region and *Siphoviridae* in the mesohaline region of the estuary. The prokaryotic abundance was high ranging from 2.7 − 3.1 × 10^6^ cells mL^−1^. Previous studies from this estuary have reported high prokaryotic abundance and our recent study demonstrated prokaryotic abundance as the best predictor variable determining the abundance of viruses in the CE^23,24,38^. Hence, it is possible that bacteriophages are the most dominant families in this estuary. Bacteriophages are the overwhelming viral types captured in metavirome investigations of water samples^39,40^. Previous reports on aquatic viromes suggested high prevalence of *Caudovirales* in the marine environments of Sargasso Sea^33^, and Iquique- Chile^34^. Recent reports from other aquatic environments also suggest a high dominance of *Myoviridae*, such as from Goseong Bay (South Sea, Korea)^41^, and some freshwater lakes^42,43^. Similar dominance of bacteriophages making up most of the viral fraction has been reported previously^11,34,35,44–46^.

In the present study, the relative contribution of the dominant families *Myoviridae*, *Podoviridae*, *and Siphoviridae* varied spatially. *Siphoviridae* formed the second dominant family after *Myoviridae* indicating the presence of temperate phages. Though some metagenomics studies report that “siphophages are the most abundant genome arrangement on earth”^11,47,48^, studies from Chesapeake Bay report on very low percentages of siphophages, especially during warm productive summer months^12^. Both sipho- and podo-phages infect a narrow range of host species^49^ and are also referred to as ‘specialist phages’. However, podoviruses were least represented in the present study, probably because of their small genome sizes. Metavirome analysis from four oceanic regions suggests that the marine viral ‘species’ are globally distributed, but the relative abundance of viral genotypes fluctuates between specific ecosystems^10^. However, the metagenomics studies during the Global Ocean Sampling (GOS) indicated that among *Caudovirales*, myoviruses are ubiquitously distributed, whereas, podo- and siphoviruses are more geographically isolated^50^. In addition, the distribution of tailed bacteriophages is also influenced by environmental factors in the world’s oceans. The abundance of podoviruses is positively correlated with salinity, whereas abundance of myoviruses was more dependent on temperature^36^.

Classification of metagenomics sequences indicated that *Synechococcus* phage was the most commonly detected phage throughout the estuary. Recent studies from CE reported the abundance of *Synechococcus* species during pre-monsoon period^51^. The ‘cyanophages’, especially *Synechoccus* phage, are more abundant in many aquatic environments^52,53^. They play significant roles by participating in the maintenance of community diversity, abundance, and seasonal succession of their hosts^54–58^ mostly by ‘killing the winner hypothesis’^59^, and through the movement of genes throughout the host population^60–62^. Phylogenetic and metagenomics studies also demonstrate the presence of endemic populations of *Synechoccus* phages from Chesapeake Bay^12^. In Chesapeake Bay, cyanophage assemblage was dominated by small-genome, narrow host range cyanopodophages.

Determination of functional activity is important for understanding and manipulating ecosystems. A wide range of molecular, biological, and cellular functions were found in viromes in the CE. The major annotated molecular functions were ATP binding and DNA binding. A recent report of viral genetic diversity from a mangrove origin found that viruses there possessed a high number of genes for molecular function, such as ATPase, single-stranded DNA-binding protein, DNA ligases, helicase and several nucleases; these functions were required for viral replication inside the host cell^63^. The environment in which the organisms live determines the metabolism or functional activity of viruses in that particular environment. Most of the viral functional diversities are similar for all the communities, but their relative occurrence varied based on the biogeochemical conditions of the environment^19^. One of the main biological functions performed by viruses in the CE included photosynthetic electron transport in photosystem II. Many studies have reported on the presence of photosystem II core reaction center protein DI, encoded by *psbA* gene in marine cyanophages^12,61,64^. *Psb A* encoding genes are known to be transcribed during lytic infection^65^. It is reported that 88% of the cyanophages in Chesapeake Bay carry *psbA* gene mainly for maintaining host photosystem functionality during infection. The susceptibility of cyanophages to carry these genes concur with the host specificity and/or genome size of a given strain^62^. Both broad- host-range cyanomyoviruses and narrow host range cyanopodo- and cyanosipho-viruses possess photosystem II core reaction center protein.

## Conclusion

Metavirome sequencing from the Cochin estuary provides a fundamental insight into the viral diversity in a highly productive tropical monsoonal estuary. Our study demonstrated that the most dominant family in this estuary were *Myoviridae*, *Siphoviridae*, and *Podoviridae*. Functional predictions of viral proteins suggested important molecular, cellular and biological functions such as ATP binding, DNA binding, ATPase, DNA polymerase, hydrolase, helicase activity, endo and exo nuclease activity, DNA repair, DNA integration, and photosynthetic electron transport in photosystem II. However, a large percentage of viral sequences were unclassified especially in the freshwater region, PR4 of the estuary. Our study also demonstrated spatial variability in the relative abundances of dsDNA viruses in relation to the different salinity regimes of the estuary. This data is immensely valuable in enhancing our understanding about viruses in a tropical highly productive estuarine environment. However, the results of this study are limited by only dsDNA viruses (LASL method) and large amount of unknown sequences without any similarity with the known sequences in the database. Future studies in CE must include additional approaches to target ssDNA and RNA viruses.

## Materials and Methodology

### Study site and sampling

Cochin estuary (CE) is an oxbow-shaped and one of the largest tropical estuaries in India (256 km^2^). It receives ~2 × 10^10^ m^3^ year^−1^ of fresh water from six rivers (Periyar, Pamba,Achankovil, Manimala, Meenachil and Muvattupuzha) and salinity incursion from the Arabian Sea^66^. The estuary opens to the Arabian Sea through two inlets- Munambam inlet (150 m wide) and the Cochin inlet (450 m) **(**Fig. 1). The annual rainfall of the region is around 320 mm, of which nearly 60% occurs during the southwest monsoon (June–September). During the pre-monsoon season (February–May), the increased tidal activity modifies the flushing characteristics of the estuary^67^. The average tidal range of the estuary is 1 m. During this season high saline waters from the Arabian Sea enter the estuary through the inlets and lower reaches of the estuary acts an extension of the Arabian Sea^68^. Along the entire stretch of CE, water quality varies depending upon the region-specific human activities. Previous studies on biodiversity suggest that the species diversity, richness and evenness were high during the dry pre-monsoon season in CE^25,26^. This could be due to reduced freshwater flow during the pre-monsoon season resulting in a warmer estuary with reduced turbidity (due to low run off) and high solar radiation, eventually facilitating high biological production. Accordingly, samples were collected during pre-monsoon from four stations (Supplementary Fig. 1). Station PR1 was located at the Cochin inlet which represented a highly dynamic region receiving high saline waters from the Arabian Sea. Station PR2 was located ∼8 km to the south of the inlet, where lot of industrial wastes are released. Station PR3 was located on the northern side of the estuary adjacent to the vast area of aquaculture activities, whereas station PR4 was located at the southern end of the estuary which receives substantial amount of agricultural wastes. Water samples were collected from 0.5 m depth using Niskin water samplers in the month of March, transferred to sterile acid-washed bottles, and brought to the laboratory within 2 hours of collection to be analyzed for various parameters.

### Environmental parameters

The physicochemical parameters such as temperature and salinity were measured using a conductivity temperature density profiler (SBE Seabird 19 CTD, Seabird Scientific, USA) with accuracy of ±0.001 °C for temperature and ±0.001 S/m for conductivity. Salinity was also measured using an Autosal (Guild line) for correcting the CTD salinity. The CTD profiler is pre-calibrated and calibrated periodically by the manufacturer, Seabird. Water samples were brought to the laboratory within one hour of collection and analyzed for dissolved inorganic nutrients, such as nitrate (NO_3_-N), nitrite (NO_2_-N), ammonia (NH_4_-N), phosphate (PO_4_-P), and silicate (SiO_4_-Si), spectrophotometrically following standard procedures^69^. The dissolved oxygen (DO) was estimated by Winkler’s method. Chlorophyll *a* was measured by filtering 500 ml water samples through GF/F filters. The pigments concentrated on the filters were extracted with 90% acetone for 24 h in the dark at 4 °C^70^, and the fluorescence was measured using a fluorometer (Model 7200-000, Turner Designs, Trilogy, USA). The fluorometer was calibrated using known standards, twice a month (Sigma, USA).

### Enumeration of viruses (VA), prokaryotes (PA) and Total viable count (TVC)

Water samples collected using 5-liter Niskin bottles were immediately transferred into 50-ml centrifuge tubes and stored on ice; prior to microscopy, these samples were fixed with formaldehyde (final volume, 2%). Viral particles and bacterial cells were filtered from 1 mL of water sample by gentle vacuum filtration onto a 25-mm diameter, 0.02 μm pore-size Anodisc (Whatman) and stained with SYBR green I fluorescent dye (Invitrogen, CA, USA) as previously described^71^. The filter was air dried on absorbent paper and mounted between a slide and a glass coverslip with a special antifading mountant [50% glycerol, 50% PBS - phosphate buffered saline (0.05 M Na2HPO4, 0.85% NaCl, pH 7.5), 0.1% p-phenylene diamine]. When not analyzed immediately, slides were stored at −20 °C until counting under an epifluorescence microscope (Olympus BX 41, Olympus, Japan). Prokaryotes were distinguished from virus-like particles (VLPs) on the basis of their relative size and brightness^71^. A blank (sterile 0.02 μm filtered double distilled water), was routinely examined as a control to check for contamination of the equipment and reagents.

TVC was measured to estimate the physiologically active bacteria^72^. Briefly, 5 ml of water sample was mixed with 50 µl of 0.05% yeast extract and 50 µl of an antibiotic cocktail (nalidixic acid, pipemidic acid, piromidic acid, and cephalexin). After incubation in the dark for 6 hours, the samples were fixed in 2% formalin, filtered through 0.2 µm pore-sized 25 mm diameter black nucleopore filter (Whatman), stained with 100 µl of acridine orange (0.1 g/100 ml), and enumerated using an epifluorescence microscope.

### Viral concentration and processing

A 200-L of water sample was collected using a 10 L Niskin sampler (operated multiple times) to concentrate the viruses for viral metagenomic analysis. Briefly, the water samples were prefiltered through a 5-micron nitex mesh and concentrated to approximately 300 ml using a tangential flow filter (TFF) (CDUF001LT-Millipore, 30-kDa cut off). During filtration, pressure was kept below 0.6 bar (10 psi) to ensure that the microbial cells were not destroyed. The samples were stored at 4 °C until further processing^12,34,73^.

### Sample processing for DNA isolation and sequencing

The TFF viral concentrates were filtered through a 0.22 µm sterivex filter (Millipore, USA) to remove any bacterial contamination. The viral fractions were treated with DNase I (20 U/ml at 37 °C for 30 minutes) to eliminate free DNA. DNase-treated samples were further concentrated using a centrifugal concentration filters (Amicon Ultra, Millipore, USA) before DNA extraction. The DNA extraction was performed using DNeasy Power Soil Kit (Qiagen, Germany) as per the manufacturer’s instructions. The metagenomic DNA was quantified using a genomic DNA quantification kit and purity was determined using a Nanodrop Spectrophotometer (Nanodrop 2000, Thermofischer Scientific, USA). The integrity of genomic DNA was verified on a 0.8% agarose gel (Sigma-Aldrich, USA) and the gel image was documented using Multi Doc-IT^TM^ Imaging System (Ultraviolet products Ltd, Analytik-jena, USA). DNA was stored at −20 °C until further downstream processing (https://www.uvp.com/manuals/81021401.pdf). Potential contamination due to prokaryotic and eukaryotic DNA in the viral DNA samples was verified by PCR targeting 16S and 18S rRNA genes. Samples which passed this quality check were subjected to further sequencing analysis.

### NGS library preparation

Next generation sequencing (NGS) libraries were prepared by Illumina HiSeq paired-end sequencing by the linker amplified shotgun library (LASL) method using an Illumina-compatible NEXTflex Rapid DNA sequencing kit which targets only dsDNA viruses (BIOO Scientific, Texas, U.S.A.) at Genotypic Technology Pvt. Ltd., Bangalore, India. Briefly, genomic DNA was sheared using Covaris S2 sonicator (Covaris, Massachusetts, USA) to generate approximate fragment size distribution from 150 bp to 400 bp. Fragment size distribution was checked on Agilent 2200 Tape Station and subsequently purified using High Prep magnetic beads (MagBio Genomics, Inc.USA). The purified fragments were end-repaired, adenylated and ligated to Illumina multiplex barcode adaptors as per NEXTFlex Rapid DNA sequencing kit protocol^74^ http://www.biooscientific.com/Next-Gen-Sequencing/Illumina-DNA-Library-Prep-Kits/DNA-Seq.

The adapters used in the study were the Illumina Universal Adapter: 5′ AATGATACGGCGACCACCGAGATCTACACTCTTTCCCTACACGACGCTCTTCCGATCT-3′ and Index Adapter: 5′-GATCGGAAGAGCACACGTCTGAACTCCAGTCAC [INDEX] ATCTCGTATGCCGTCTTCTGCTTG-3′. The adapter-ligated DNA was purified using High Prep beads. The resultant fragments were PCR amplified for 12 cycles using Illumina-compatible primers provided in the NEXTFlex Rapid DNA sequencing kit. The final PCR product (i.e. sequencing library) was purified with High Prep beads, followed by library quality control check. The sequencing libraries were quantified by Qubit fluorometer (Thermo Fisher Scientific, MA, USA) which yielded a concentration range of about 1.8–2.5 ng/µl. The library fragment size distribution was analyzed on Agilent 2200 TapeStation (Illumina, USA) which showed a range of 200–700 bp for all libraries^74^.

### Illumina sequencing

The sequencing libraries were molar-normalized and then pooled into a single tube. The pooled sample was then diluted to 4 nM final concentration using resuspension Buffer (RSB – Illumina, CA, USA). The sample was denatured for 5 minutes using 0.2 N NaOH and neutralized by HT1 Buffer (Illumina, CA, USA). It was then pooled with other libraries prepared for NGS in a ratio dependent on amplicon size/total panel size, desired sequencing depth, and the number of samples pooled in each sub-library. Pooled libraries were further diluted down to a final 12 pM library. Samples were then loaded into an Illumina HiSeq cartridge (Illumina, CA, USA) and run in 2*150 mode on an Illumina HiSeq next generation sequencer (HiSeq. 4000 sequencer, Illumina,CA,USA) (https://www.illumina.com/content/dam/illuminamarketing/documents/products/datasheets/hiseq-3000-4000-specification-sheet-770-2014-057.pdf).

### Initial processing of sequence reads

Demultiplexing was completed using bcl2fastq Conversion Software that was embedded in the HiSeq. The automated FASTQC Tool Kit application on Illumina Base Space Labs was used to filter out quality reads, in which quality reads above Q30 were kept for downstream analysis (https://www.illumina.com/documents/products/technotes/technote_Q-Scores.pdf).

### Data analysis

The raw reads with adapter sequences and low-quality bases were removed using ABLT perl script (proprietary tool of Genotypic Technology Pvt. Ltd) to trim adapter sequences, low quality bases (phred score <30) and blocks of Ns. The processed high-quality reads (with more than 75% bases having phred score greater than 30 were considered significant for further downstream analysis. The high-throughput metavirome sequencing analysis was computed using the MetaSPADES program. Paired-end reads were assembled using the MetaSPADES-3.7.1 assembler^75^. Based on the denovo assembly, contigs with length >=300 bp were first aligned against the Refseq bacterial genome database and the unaligned contigs were aligned against NCBI RefSeq viral genome sequences (release version 80) using GBLASTN with 80% sequence identity and 85% average query coverage with an E-value cutoff of 10^−5^ for taxonomic classification^41,76,77^. The unaligned sequences against viruses were further aligned against archeal and fungal Refseq genome sequences downloaded from NCBI with the same release version and with similar cutoff parameters to ensure that only viral sequences were considered for analysis.

### Functional diversity of estuarine viruses

The contigs with length >=300 bp were used for viral protein prediction using MetaGeneMark prediction software^78^. The predicted proteins were homology searched using BLAST based method against a viral specific protein sequences obtained from the Uniprot database and were annotated with a minimum sequence identity 30% (30–100%), e-value cutoff of 10^−3^ with an average sequence coverage of 50%^79–81^.

### Statistical analysis

A one-way ANOVA was used to understand significant variations in biological parameters with respect to stations. Redundant analysis (RDA) was used to elucidate the interrelationships between the viral components and their environmental variables. Initially, the data were processed using detrended correspondence analysis (DCA) to select the suitable ordination technique. DCA resulted in an axis gradient length of <2, suggesting that linear multivariate RDA was suitable for the present data^82,83^, with species correlation scaling as ordination scores. The biological variables were log transformed prior to the analysis. Partial RDA was carried out to identify the environmental parameters contributing more to the explained variation in the biological components. The ordination significance was tested with Monte Carlo permutation tests (499 unrestricted permutations) (p < 0.05). The results of the RDA are presented in the form of triplots with stations as points and environmental variables by arrows^83^.

Venn diagram was generated for all identified taxa’s across the samples and the common and the unique organisms were reported. The Bray-Curtis dissimilarity distance matrix (https://docs.scipy.org/doc/scipy/reference/generated/scipy.spatial.distance.pdist.html) was used to generate principal coordinate analysis (PCoA) plots using QIIME^84^. A clustered row-wise heatmap was generated using the R package NMF, for all the identified species across the samples based on their relative abundance values. The color slab was generated based on the maximum and minimum values in the matrix. Cochin Estuary metaviriome were compared to previously published globally distributed metaviromes. These included datasets from different oceans, namely, Atlantic ocean (MG RAST id; 4722276.3 to 4722285.3), Gulf of Mexico (4440304.3, 4441623.3 to 4441629.3), Salton Sea (4440327.3, 4440328.3), Sargasso Sea (4441624.3, 4440322.3), Arctic sea (4440306.3), Line Islands 4440036.3, 4440038.3, 4440040.3, 4440280.3), Western Sea, Korea (4464802.3, 4464804.3, 4464805.3), Indian Ocean (4722282.3), Red Sea (4722283.3) bays (4440102, 4440330.3, 4440102.3), and Fish pond (4440424.3, 4440412.3, 4440439.3, 4440414.3). We took the top 50 identified identified taxa at family level from the public dataset (available in MG-RAST) and compared with our identified taxa’s for PR1 to PR4 samples. A common master table of the identified families was prepared and their corresponding values were fetched. PCA was plotted using R package ggplot2 using log-based normalization on these corresponding values with princomp function. (http://metagenomics.anl.gov/)^85^.

### Nucleotide sequence accession number

The sequence data from this study was submitted to the NCBI Sequence Read Archive (SRA) under accession number, SUB2990896.

## Electronic supplementary material

Supplementary figure
