# Supplementary material for: Genetic and functional diversity of double-stranded DNA viruses in a tropical monsoonal estuary, India
Source: Sci Rep. 2018 Oct 30;8:16036. doi: 10.1038/s41598-018-34332-8 (PMC6207776; doi:10.1038/s41598-018-34332-8)
Supplement: Supplementary file 1 — Supplementary figure [file 41598_2018_34332_MOESM1_ESM.pdf]

Genetic and functional diversity of double-stranded DNA viruses in a tropical monsoonal estuary, India

Jasna Vijayan[1], Parvathi Ammini[1], Abhinandita Dash[2]

[1]CSIR-National Institute of Oceanography, Regional Centre, Kochi-682 018, India

[2] Genotypic Technology, Bangalore - 560094, India

Supplementary information

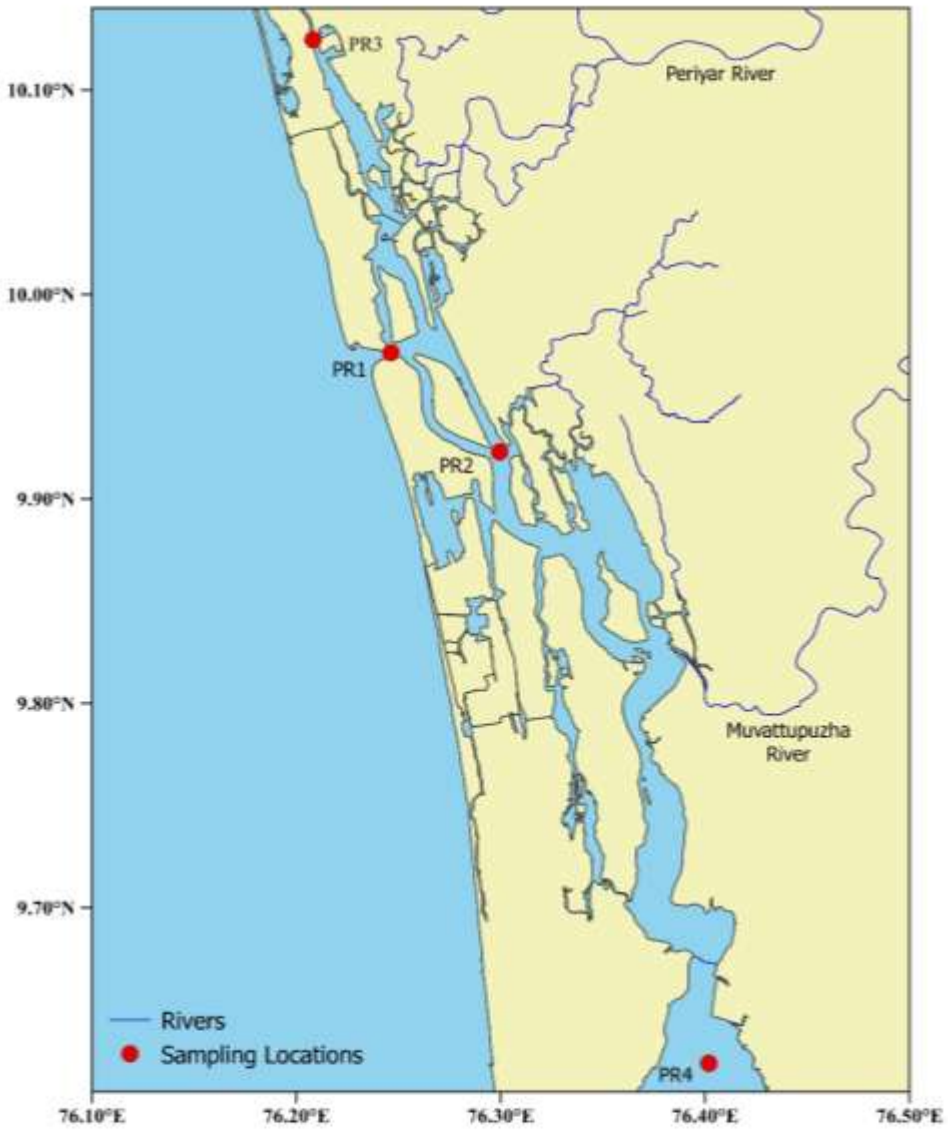

Supplementary Figure 1. Map showing sampling stations in Cochin estuary (CE). Station PR1 is the inlet. Station PR2 and PR3, represent the mesohaline region and station PR4 represent the freshwater region of the CE. The station map was created with the tool QGIS (version 2.18, Quantum GIS Development Team, 2017. Quantum GIS Geographic Information System). Quantum GIS Development Team, 2017. Quantum GIS Geographic Information System. Open Source Geospatial Foundation Project. "http://qgis.osgeo.org"). The legends included in the map such as rivers (Made with Natural Earth. Free vector and raster map data @ naturalearthdata.com,) (http://www.diva-gis.org/).

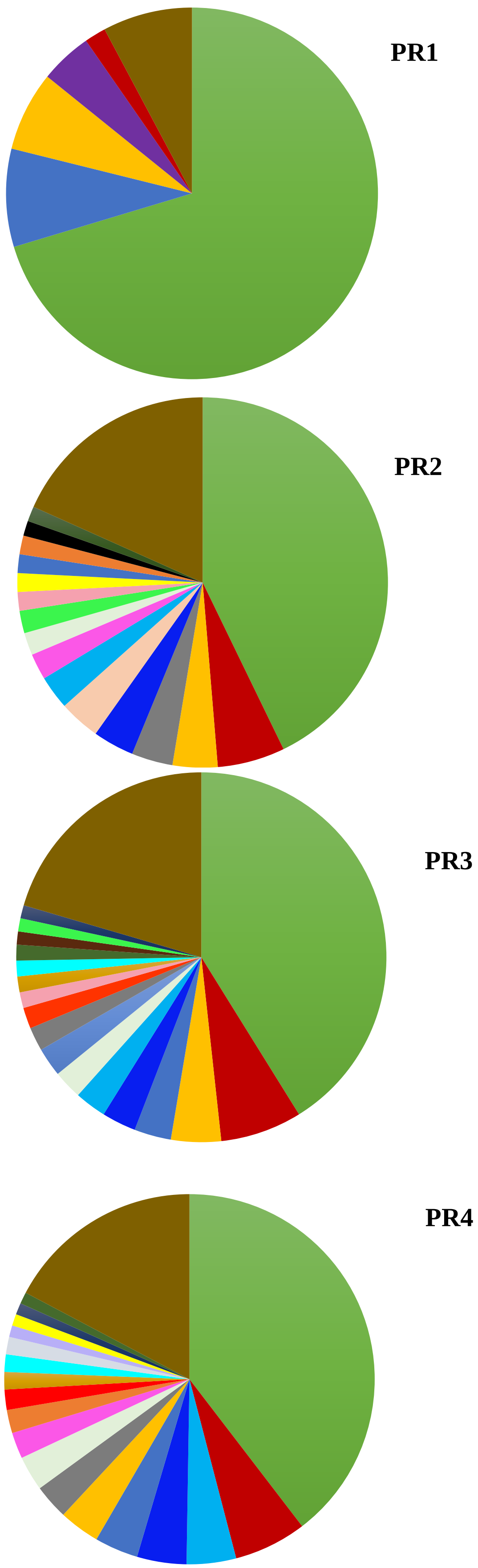

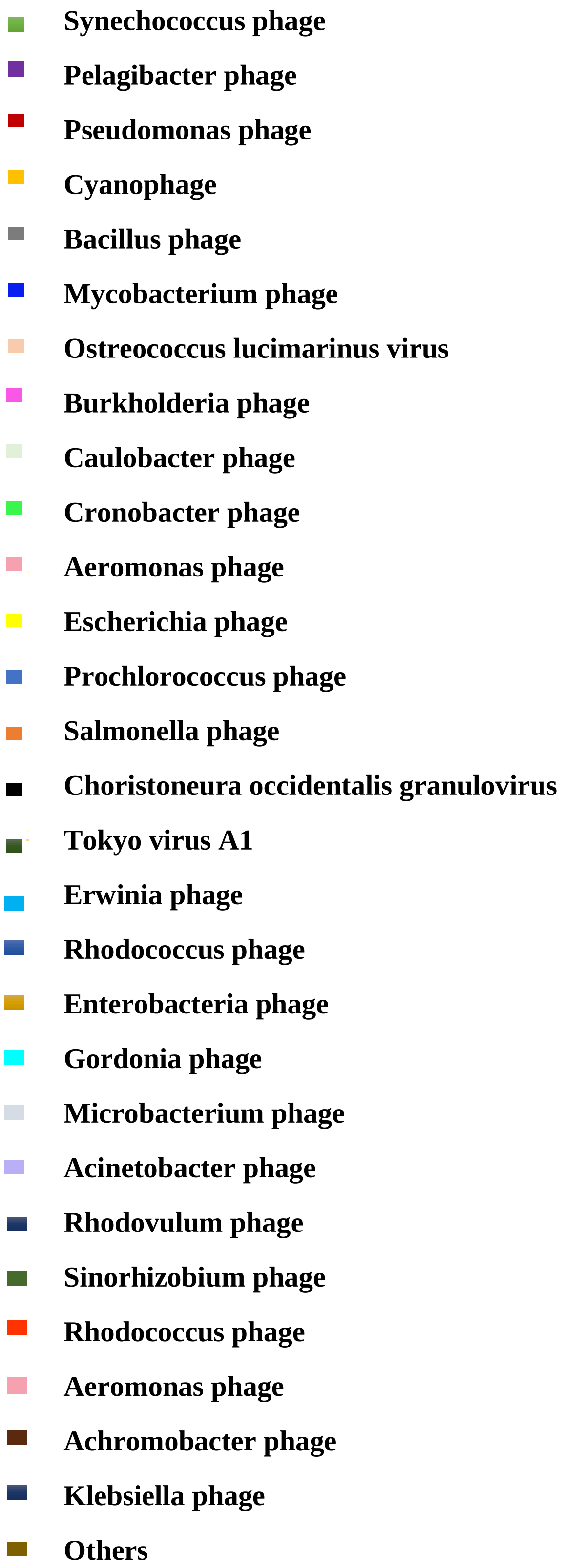

Supplementary Figure 2. Pie chart representing the spatial variations in viral species at four stations PR1, PR2, PR3 and

(3A)

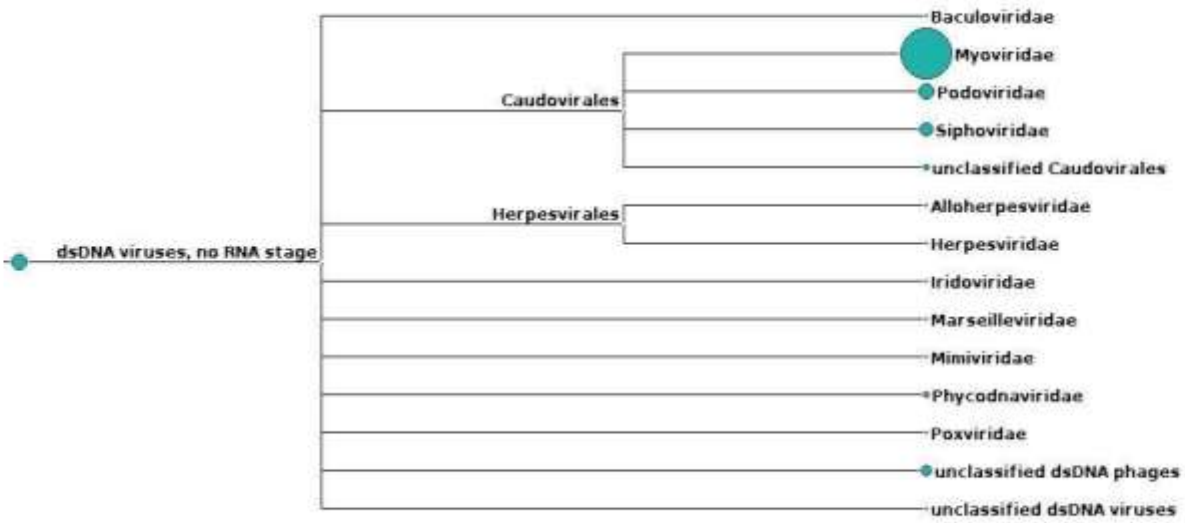
dsDNA viruses, no RNA stage
Caudovirales
Herpesvirales
Baculoviridae
Myoviridae
Podoviridae
Siphoviridae
unclassified Caudovirales
Alloherpesviridae
Herpesviridae
Iridoviridae
Marseilleviridae
Mimiviridae
Phycodnaviridae
Poxviridae
unclassified dsDNA phages
unclassified dsDNA viruses

(3b)

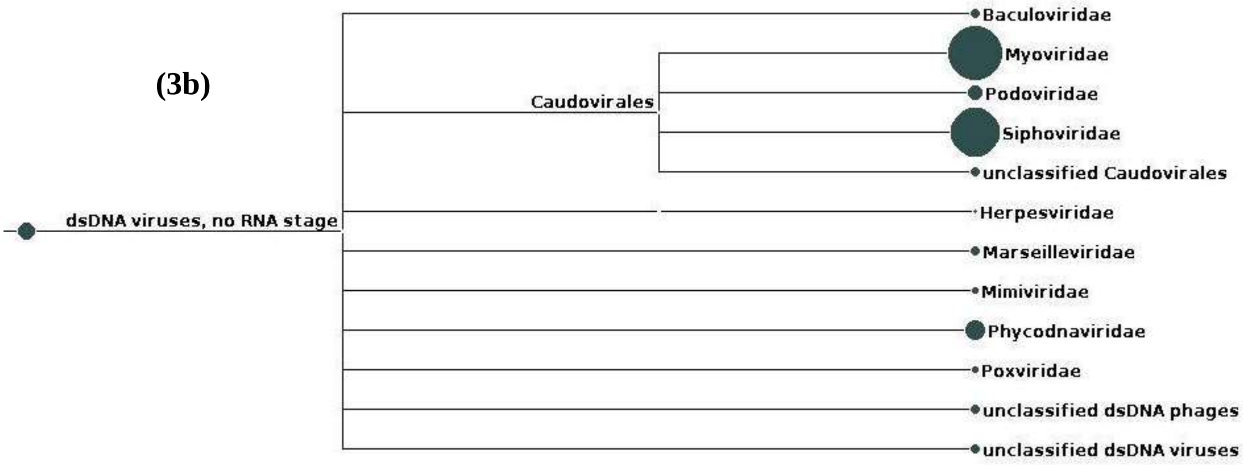
dsDNA viruses, no RNA stage
Caudovirales
Baculoviridae
Myoviridae
Podoviridae
Siphoviridae
unclassified Caudovirales
Herpesviridae
Marseilleviridae
Mimiviridae
Phycodnaviridae
Poxviridae
unclassified dsDNA phages
unclassified dsDNA viruses

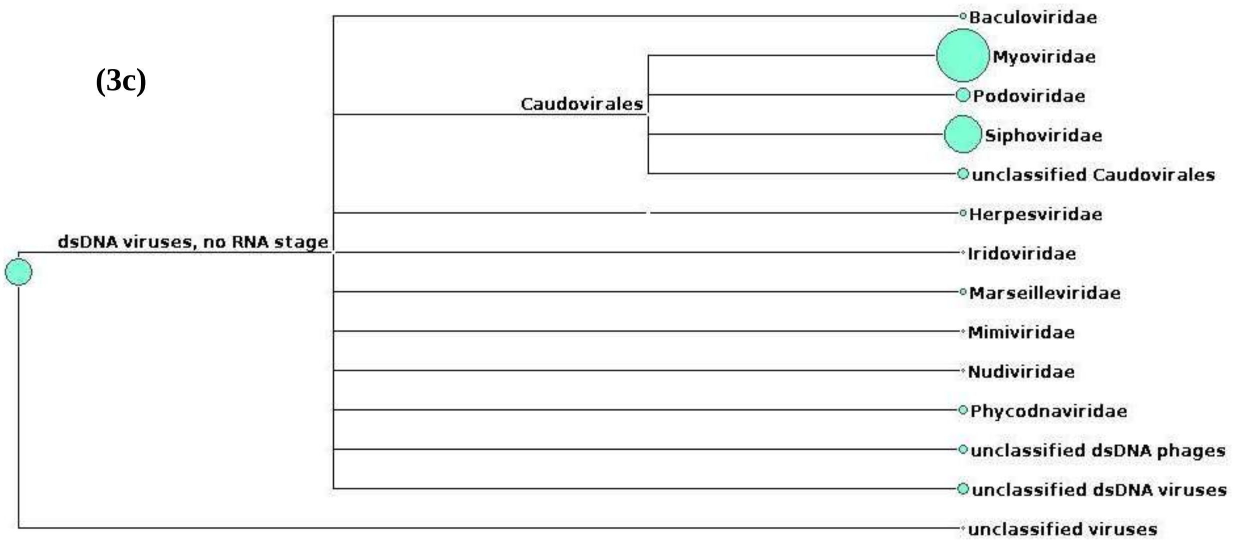

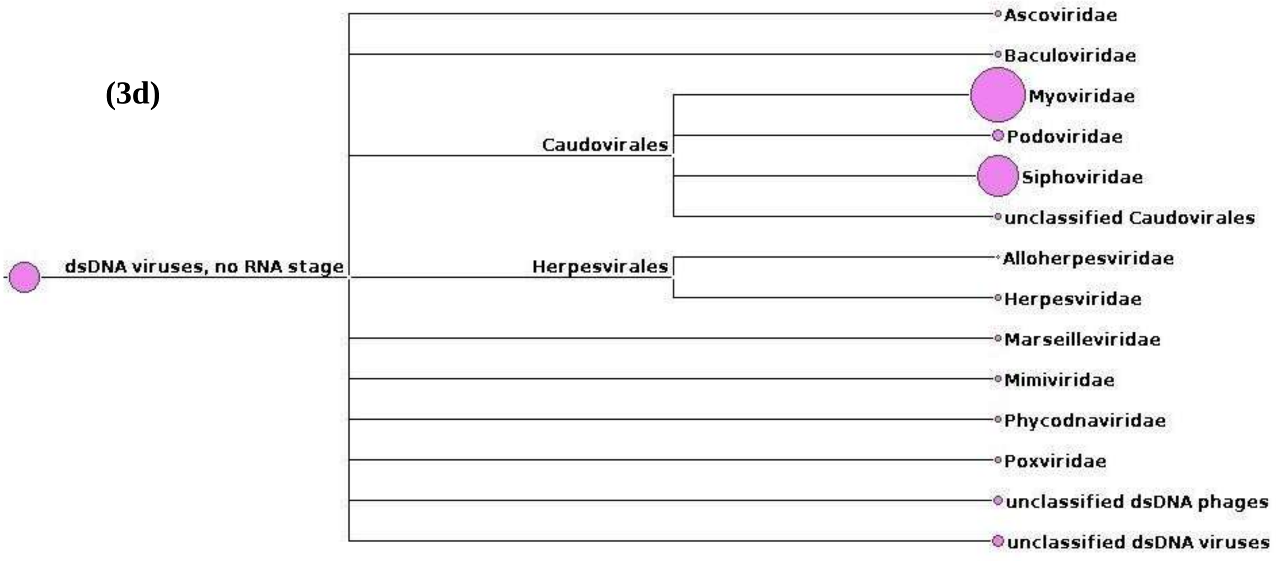

Supplementary Figure 3. Phylogenetic tree of viral families from four different stations. The panels a, b, c and d represent the stations PR1, PR2, PR3 and PR4 in the Cochin estuary.

(4a)

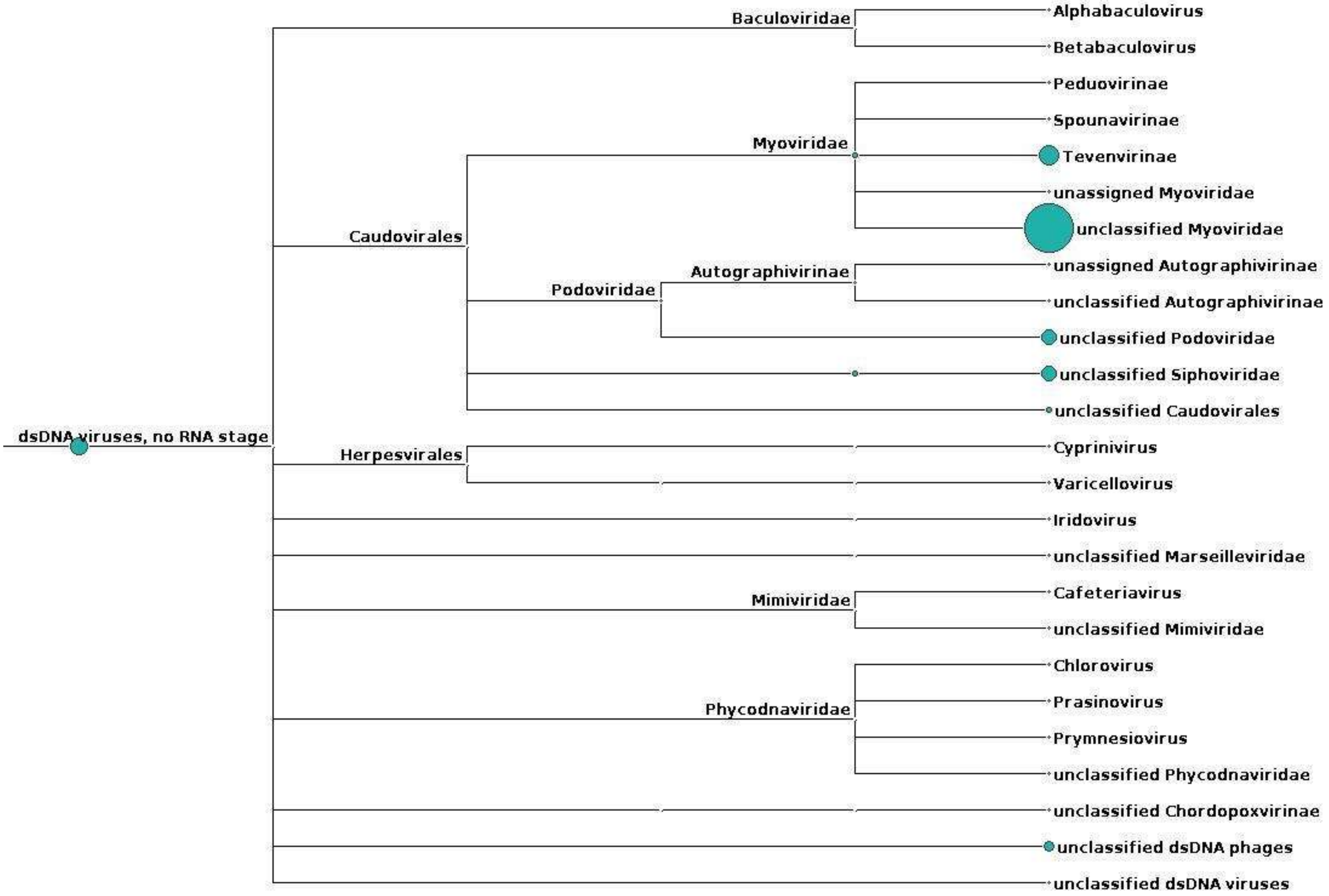

dsDNA viruses, no RNA stage
Baculoviridae
Alphabaculovirus
Betabaculovirus
Caudovirales
Myoviridae
Peduovirinae
Spounavirinae
Tevenvirinae
unassigned Myoviridae
unclassified Myoviridae
Podoviridae
Autographivirinae
unassigned Autographivirinae
unclassified Autographivirinae
unclassified Podoviridae
unclassified Siphoviridae
unclassified Caudovirales
Herpesvirales
Cyprinivirus
Varicellovirus
Iridovirus
unclassified Marseilleviridae
Mimiviridae
Cafeteriavirus
unclassified Mimiviridae
Phycodnaviridae
Chlorovirus
Prasinovirus
Prymnesiovirus
unclassified Phycodnaviridae
unclassified Chordopoxvirinae
unclassified dsDNA phages
unclassified dsDNA viruses

(4b)

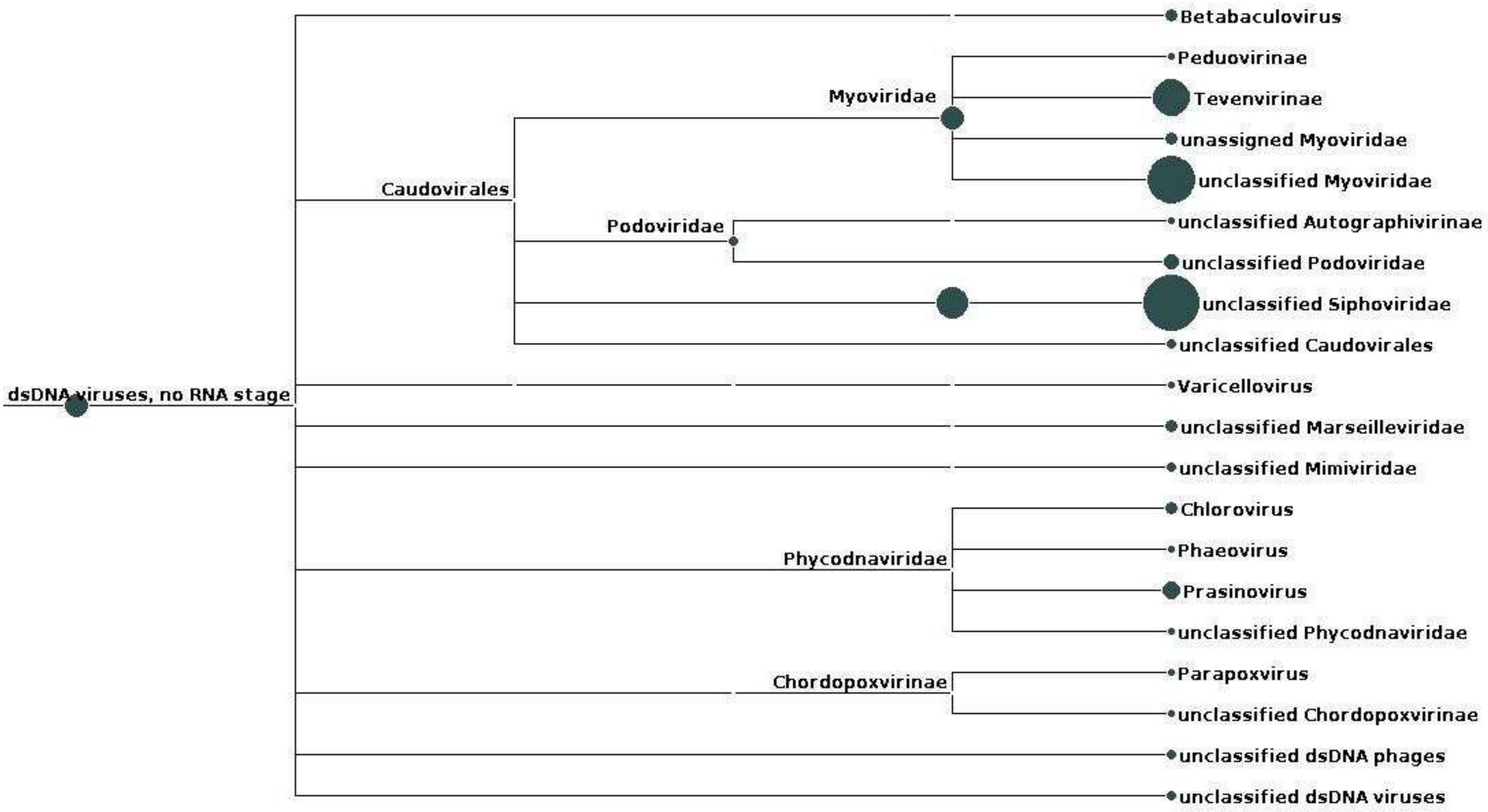

dsDNA viruses, no RNA stage
Betabaculovirus
Caudovirales
Myoviridae
Peduovirinae
Tevenvirinae
unassigned Myoviridae
unclassified Myoviridae
Podoviridae
unclassified Autographivirinae
unclassified Podoviridae
unclassified Siphoviridae
unclassified Caudovirales
Varicellovirus
unclassified Marseilleviridae
unclassified Mimiviridae
Phycodnaviridae
Chlorovirus
Phaeovirus
Prasinovirus
unclassified Phycodnaviridae
Chordopoxvirinae
Parapoxvirus
unclassified Chordopoxvirinae
unclassified dsDNA phages
unclassified dsDNA viruses

(4c)

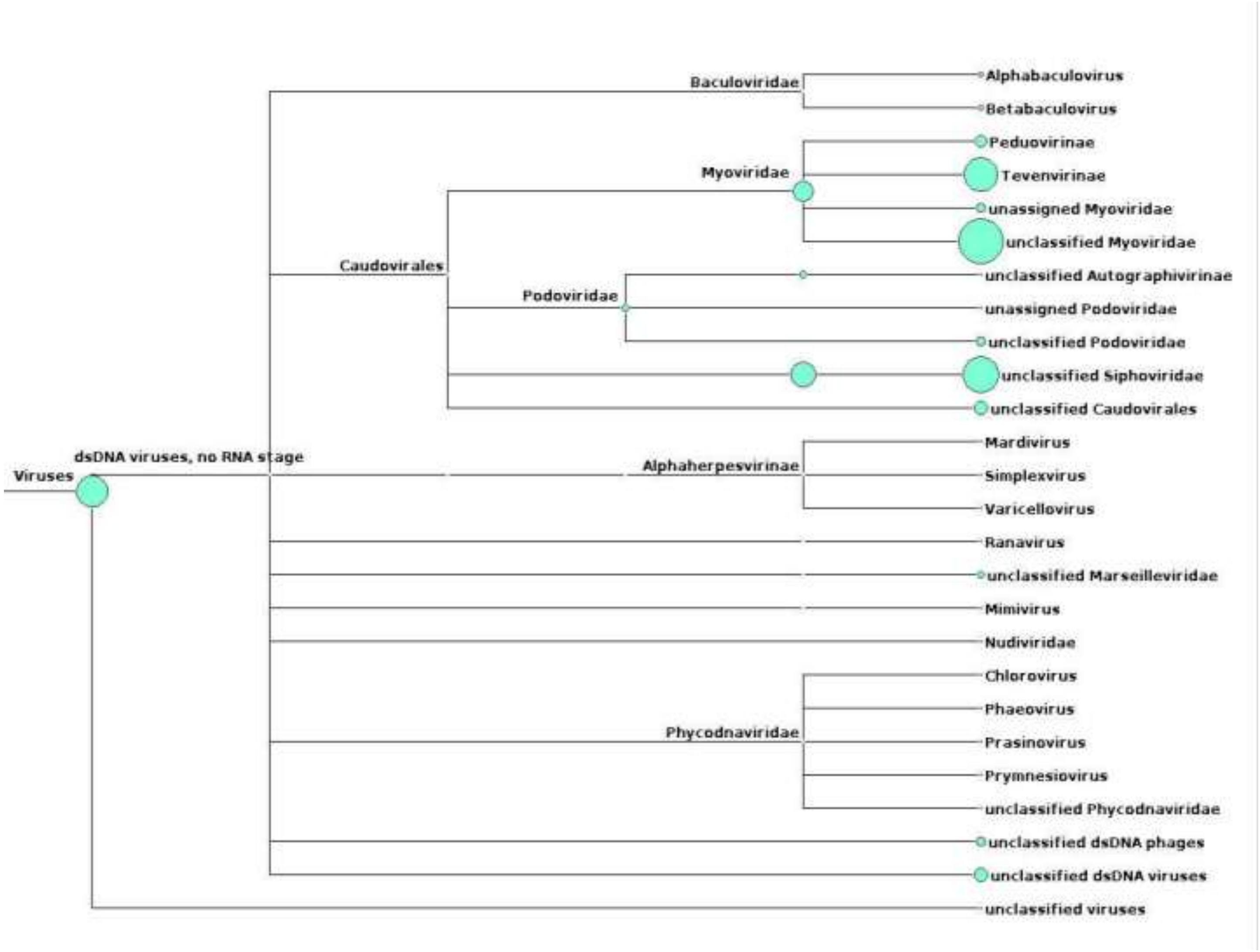
Viruses
dsDNA viruses, no RNA stage
Baculoviridae
Alphabaculovirus
Betabaculovirus
Myoviridae
Peduovirinae
Tevenvirinae
unassigned Myoviridae
unclassified Myoviridae
Caudovirales
Podoviridae
unclassified Autographivirinae
unassigned Podoviridae
unclassified Podoviridae
unclassified Siphoviridae
unclassified Caudovirales
Alphaherpesvirinae
Mardivirus
Simplexvirus
Varicellovirus
Ranavirus
unclassified Marseilleviridae
Mimivirus
Nudiviridae
Phycodnaviridae
Chlorovirus
Phaeovirus
Prasinovirus
Prymnesiovirus
unclassified Phycodnaviridae
unclassified dsDNA phages
unclassified dsDNA viruses
unclassified viruses

(4d)

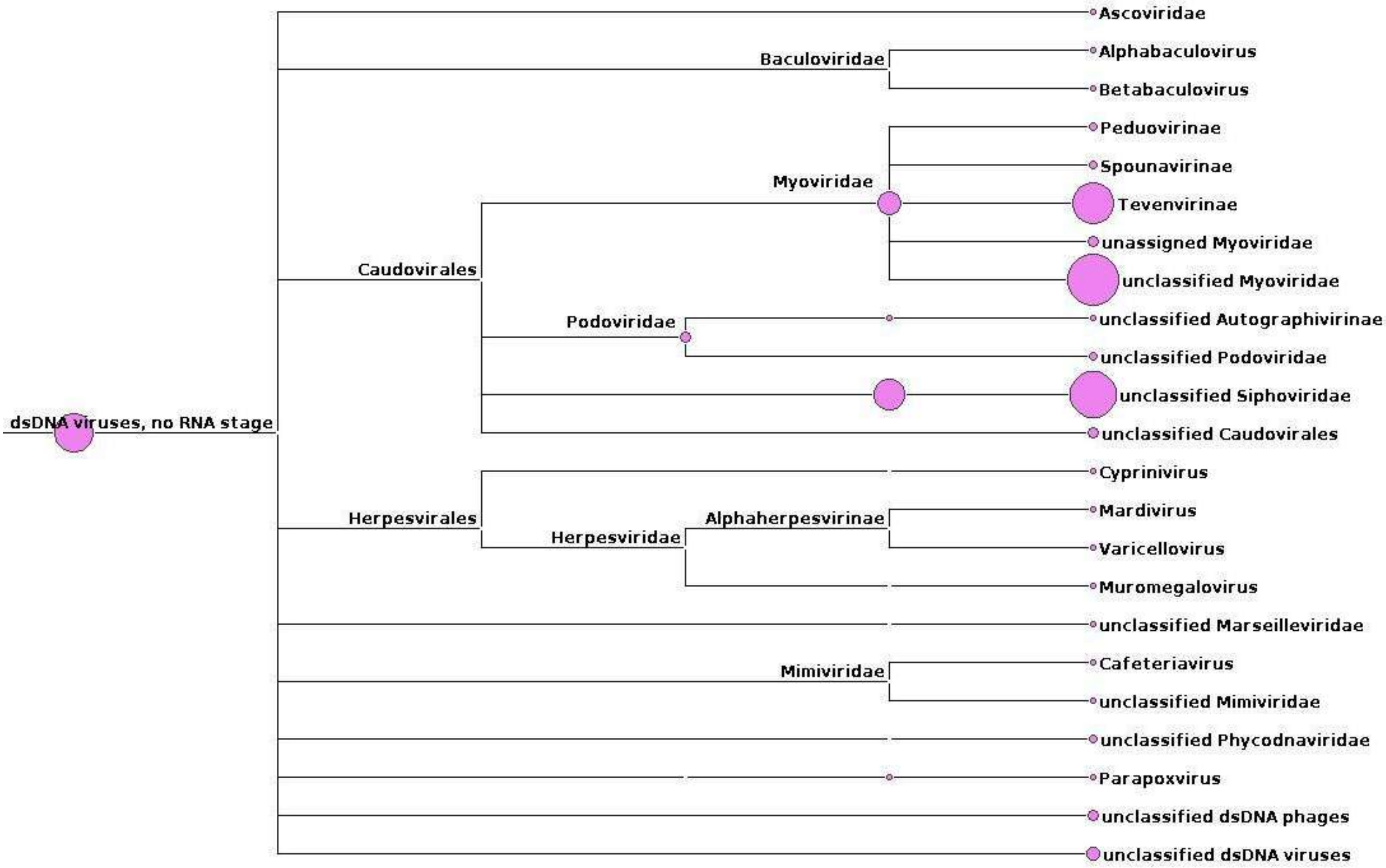

Supplementary Figure 4. Phylogenetic tree of viruses at genus level from four different stations. The panels a, b, c and d represent the stations PR1, PR2, PR3 and PR4 in the Cochin estuary.

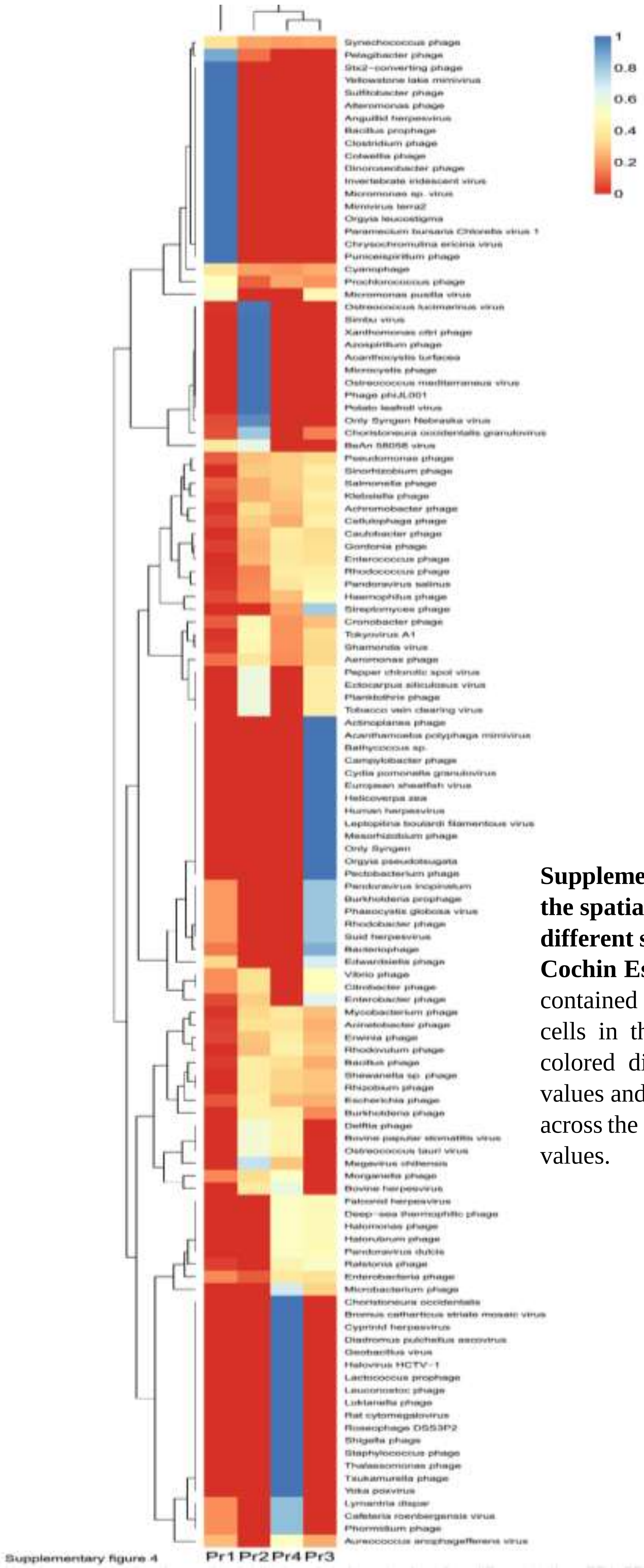

Supplementary Figure 5. Heat map representing the spatial distribution of viral species from four different stations, PR1, PR2, PR3 and PR4 in the Cochin Estuary. In heat map the individual values contained in a matrix are represented as colors. The cells in the matrix with high relative values are colored differently from those with low relative values and is generated for all the identified species across the samples based on their relative abundance values.
